# The Dual Role of Neonatal Pulse Oximetry Screening and Fetal Echocardiography in Congenital Heart Disease Detection Around the World

**DOI:** 10.3390/ijns12030055

**Published:** 2026-07-21

**Authors:** Anita Krishnan, Carolyn Sommer, Chinenyenwa Mpamaugo, Sonia Voleti Chivukula, Lisa A. Hom, Gerard R. Martin, Mary T. Donofrio

**Affiliations:** 1 Children’s National Hospital, Washington, DC 20010, USA; csommer@childrensnational.org (C.S.); cmpamaugo@childrensnational.org (C.M.); lhom@childrensnational.org (L.A.H.); gmartin@childrensnational.org (G.R.M.); mdonofri@childrensnational.org (M.T.D.); 2 George Washington University School of Medicine and Health Sciences, Washington, DC 20010, USA; 3 UCLA Mattel Children’s Hospital, Los Angeles, CA 90095, USA; svoleti@mednet.ucla.edu

**Keywords:** pulse oximetry screening, fetal echocardiography, healthcare access, congenital heart disease

## Abstract

Congenital heart disease is the most common birth defect worldwide. Perinatal detection, either in utero or prior to hospital discharge is important for planning delivery, ordering adjunct testing, and planning for surgical or medical care. In countries with access to ultrasound screening, prenatal detection is highly accurate though with variable sensitivity and specificity around the world. Pulse oximetry screening, in some cases coupled with murmur auscultation in the nursery can also reach very high degrees of sensitivity for critical congenital heart disease. Use of the modalities per guidelines and recommendations is an important aspect of decreasing infant mortality and morbidity worldwide.

## 1. Introduction

Congenital heart disease (CHD) is the most common birth defect worldwide, affecting approximately 1% of infants born each year [1]. It is beneficial to detect CHD (requiring surgical or medical management or follow-up) prior to discharge from the newborn nursery. Pulse oximetry screening efforts have focused on the identification of 13 lesions that cause hypoxemia [2]; fetal echocardiography identifies these defects as well as a broader spectrum of disease, including lesion-like septal defects and mild aortic or pulmonary stenosis, which have less severe symptoms but may unmask a genetic abnormality. In the discussion that follows we will focus primarily on these 13 lesions causing hypoxia, though in sections focusing only on fetal echocardiography, we will also include consideration of non-cyanotic but surgical disease such as double-outlet right ventricle or large ventricular septal defects.

## 2. Benefits of Prenatal Detection

The diagnosis of a congenital heart lesion triggers a cascade of interventions which are important for the survival and long-term health of affected infants; these interventions include close nutritional follow-up and supplementation, surgical care, provision of cardiac medications, neurodevelopmental assessment, and screening for genetic and extracardiac disease. Specific pathways for delivery room management and resuscitation have been described [3,4,5,6,7]. Interventions such as those for vein of Galen malformation [8] or other cardiac conditions can be obtained [9,10]. Additionally, if needed, referral to a tertiary care setting or in utero transfer can be organized [11]. Pulse oximetry screening has been a life-saving intervention for the detection of critical CHD; while legally mandated and widely implemented, patients still face barriers to access in resource-limited settings across the world.

Prenatal detection using ultrasound is another strategy for detection, though currently it only captures 2/3 of patients at most in developed nations, and significantly less, if any, in low-income countries. While both techniques have challenges in low- and middle-income countries, fetal cardiac screening using ultrasound or detailed fetal echocardiography is more challenging than pulse oximetry in these regions due to the need for subspecialists and equipment. In this article, we will discuss the current state of prenatal detection of CHD, limitations, and the ideal approach of prenatal cardiac screening, fetal echocardiography, and pulse oximetry screening working together to achieve timely, universal prenatal detection.

Significant advances in genetic testing, such as whole exome sequencing and cell-free DNA testing, which often follow a CHD diagnosis and reveal more complex holistic medical concerns, have made it more important to recognize fetal CHD. In recent years, multicenter collaborative efforts through the Fetal Heart Society and other cardiology collaboratives [12] have facilitated new knowledge and the ability to conduct more effective multicenter studies to disseminate research findings.

## 3. Global Approaches to Pulse Oximetry Screening

Various screening algorithms exist with the goal of maximizing sensitivity and disease detection while maintaining a low number of both false positives and false negatives. Most CCHD screening algorithms share similar cut-off values, requiring oxygen saturation levels above 90% to pass and a less than 4% difference between pre- and post-ductal saturations, but many have been adapted to unique regional care delivery systems including home births in the Netherlands, earlier screening to detect additional non-cardiac disease that also present with hypoxemia (such as neonatal sepsis and pneumonia) such as in the UK [13], and in countries such as Morocco [14] and Saudi Arabia [15] where routine discharge from a birth hospital may be customary less than 24 h after birth. Regional studies from China [16,17] and in other parts of the world have for some time investigated maximizing detection through combined prenatal and CCHD neonatal pulse oximetry screening with the timing of newborn physical assessment.

## 4. Recommendations Regarding Fetal Echocardiography

Detecting cardiac abnormalities before birth allows for delivery planning and critical postnatal management. In experienced hands, it is possible to diagnosis CHD as early as 12–14 weeks with high accuracy and sensitivity. McBrien et al. reported that early fetal echo has a sensitivity of 93% and specificity of 100%. In their multiyear study of 1491 patients, the positive predictive value was 100% and negative predictive value was 99.7%. Limitations were similar to typical fetal echo and included ventricular septal defects, valve anomalies, and diseases that developed in late gestation [16].

Standard guidelines for the performance of fetal echocardiography exist, focused on systematic evaluation of fetal cardiac anatomy, function, rhythm, and blood flow using two-dimensional imaging, Doppler techniques, and measurements of cardiac structure [3,4]. Since the development of these guidelines, prenatal detection of CHD in the US improved to about 66% of cases since the early 2000s. The benefits to prenatal diagnosis include improved neurodevelopmental outcomes [5], appropriate delivery planning [3], decision-making options, shorter hospital length of stay, and benefits for the family in terms of financial planning [6]. Despite these benefits, prenatal detection of CHD has remained about the same for the past ten years [7,8]. Missed prenatal diagnoses disproportionately affect poor and rural communities. A number of studies have explored the underlying system failures associated with missed prenatal diagnosis, including public insurance and/or Hispanic ethnicity in low-resource communities [7,8,9,10]. One study identified that location of the anatomic scan is an important factor in detection, and likely has differences in rural regions [2]. Therefore, targeted educational interventions in low-resource areas may improve overall detection rates.

## 5. Factors Underlying Disparities in Prenatal Diagnosis

Several studies have examined the sociodemographic and geographic factors which influence the receipt of a prenatal diagnosis of CHD. Prior studies have examined insurance status [17,18], socioeconomic position [19], and race/ethnicity [20]. In North America, regional variations exist; Hill et al. showed that rurality is associated with missed prenatal detection in a single-center midwestern study [17]. Quartermaine et al. examined surgical databases and showed that lesions requiring outflow tract scanning were less likely to be detected than those seen on four-chamber view [21]. In 2020, a multicenter research registry of over 1000 infants with hypoplastic left heart syndrome (HLHS) and d-transposition of the great arteries (D-TGA) was created. The study was unique in creating a specific dataset to study socioeconomic position using more granular census tract data and a more well-defined small number of lesions. The data revealed that missed prenatal detection was associated with low socioeconomic position (neighborhood composite score), living in a rural region, and Hispanic ethnicity, with overlap between socioeconomic status (SES) and rurality [20]. These factors also led to delays in timing of prenatal diagnosis, even in those who received a diagnosis. The study was retrospective and corroborated by further work post-pandemic through the Fetal Heart Society, showing that 1/3 of patients undergoing surgery did not receive a prenatal diagnosis [22]. Even in heavily resourced areas, deserts of subspecialty care exist. Obstetric care shows significant deserts in rural regions, with vast swaths of the country lacking access to nurses, midwives, delivery hospitals, and high-risk obstetric care. Referral patterns may be entrenched in certain places due to lack of trust or to financial costs associated with some private practice providers that family practice doctors and midwives may not want to incur for their lower-resourced families. Additionally, the prevalence of home births may necessitate new considerations for pulse oximetry screening, even if midwives are skilled in this technique and can provide the screening.

Worldwide, CHD screening is based on routine obstetric ultrasound and not on fetal echo that is performed by fetal cardiologists as no country has adequate numbers of fetal cardiologists to screen their respective populations. Both the Fetal Heart Society and the International Society of Ultrasound in Obstetrics and Gynecology (ISUOG) acknowledge that screening and detection of CHD varies depending on equipment availability and operator expertise. Therefore, ISUOG guidelines have explicitly focused on the evaluation of cardiac anatomy during routine obstetric ultrasound. The new guidelines suggest that reliance on the four-chamber view alone misses many serious CHDs, despite many low-resource screening programs relying solely on the four-chamber view. Detection rates of CHD can be improved by including examination of the outflow tracts. This additional screening implementation requires task-shifting models, such as training midwives or general ultrasound technicians. Internationally, countries who implemented training programs for anomaly screening have shown improvement in detection rates [13,14].

## 6. How Does Prenatal Diagnosis of CHD Improve Neonatal Outcome?

Prenatal diagnosis decreases morbidity and mortality in critical CHD [23,24], and has benefits for families even when lesions are non-critical [25]. The benefits of prenatal diagnosis include perinatal stabilization, coordination of timely interventions such as catheterization [3], and selection of a delivery center in proximity to the tertiary care center [3]. Unfortunately, prenatal diagnosis even for the severest forms of CHD occurs in only 2/3 of cases in the US; in other countries such as the Netherlands less than half are detected [12,22,26], and this may be an overestimate depending on the lesion and region [20]. Families living in poverty have unique economic stresses which may make prenatal planning more important [25]. Prenatal detection of CHD also has benefits related to neurodevelopmental outcomes and may correlate with differences in structural brain alterations [27].

Once diagnosed, fetuses with CHD receive closer follow-up [4] and delivery management planning. They also may be eligible for additional close follow-up by high-risk obstetricians. Intra-uterine growth restriction is common in fetuses affected by CHD [28], and low birthweight and prematurity remain the leading infant factors in surgical mortality. Timely prenatal diagnosis of CHD can prompt referral to a high-risk obstetrician to mitigate these co-existing risk factors and a pediatric cardiologist to closely surveil for changes in cardiovascular hemodynamics which warrant therapy. Fetuses with a prenatal diagnosis of CHD typically receive more frequent surveillance in the third trimester with a maternal fetal medicine specialist, bringing benefits to both mother and baby. Pre-eclampsia [29] and growth restriction are more common in pregnancies complicated by CHD in the fetus, further highlighting the need of early diagnosis and co-management with maternal fetal medicine specialists.

The field of fetal cardiology began rapidly evolving in the early 2000s, less than 25 years ago. Prior to that time, the diagnosis rates were quite low, and infants were admitted to intensive care units in extremis as a routine event. The ability to intervene in the perinatal period has brought significant benefits, and is still evolving [3,5,6,7]. Attention to maternal mental health and well-being as determinants of fetal brain development are also increasing. Additionally, with careful meticulous follow-up, subtle changes in blood flow, heart function, or rhythm may warrant interventions such as specialized delivery planning [30], initiation of medications or arrhythmia management [29], rapid pacemaker implantation, and fetal interventions [31]. In the absence of prenatal diagnosis, innovative techniques and clinical trials cannot be offered. Therefore, early prenatal diagnosis is important for ensuring innovation in the field.

## 7. International Studies Comparing Prenatal Echocardiography and Pulse Oximetry Screening

Clinicians have debated whether obstetrical ultrasound cardiac screening and fetal echocardiography (a more specialized test) have eliminated the need for pulse oximetry screening after delivery. Current AAP and WHO guidelines emphasize the unique benefits of both prenatal and postnatal screening tests and recommend pulse oximetry screening worldwide, even when the obstetrical ultrasound or fetal echocardiogram is normal. Additionally, pulse oximetry screening captures respiratory illness, anemia, sepsis, and a number of other non-cardiac conditions which cause hypoxemia. Data from the International Clearinghouse for Birth Defects Surveillance and Research [32] have shown that international prenatal detection of critical CHD is approximately 50%, but ranges from 13% (Slovak Republic) to 87% (France). Other studies describe sensitivity as high as 98% in France for severe disease [33]. Certain lesions like total anomalous pulmonary venous drainage had very low rates of detection (28%) in that series, which importantly included countries where anatomic scanning by ultrasound is available to all women. Interestingly, countries with low detection rates (Argentina, Malta) had increased neonatal mortality. Even in the US, two centers (Arkansas and Atlanta) had dramatically different prenatal detection, with Arkansas being quite low. When studying the use of pulse oximetry with other forms of prenatal screening, a major study of over 20,000 infants in the UK found that for infants who received antenatal screening in the absence of suspicion of CHD, pulse oximetry was 58% sensitive for detecting critical cases [34]. When extrapolated to a population of 100,000 babies assuming a 50% prenatal (antenatal) detection rate, pulse oximetry would identify an additional 35 cases of critical CHD that were not detected before birth. These benefits are presumed to be even greater in areas with lower prenatal detection rates. Data has shown that pulse oximetry adds value to the current screening methods for the identification of critical CHD that would otherwise go undetected. Further, while access to fetal echocardiography continues to be limited, especially in under-resourced countries, pulse oximetry can be a significant benefit for the post-birth detection of critical CHD.

## 8. Global Screening for CHD

Prior publications have shown that CHD screening using pulse oximetry was adopted earlier by Nordic countries, the UAE, and European countries, and lagged in Africa and South and Central America. Similarly, ultrasound-based screening for CHD is infrequently performed in middle-income countries, and formal fetal echocardiography is performed less than 50% of the time even when CHD is suspected [35]. In China, Li et al. described a statistically significant improvement in using pulse oximetry screening in combination with auscultation for a murmur in detecting CHD in a cohort of over 3000 infants. This was termed the “joint index method”. Sensitivity was 100%, as was negative predictive value [36]. The strategy of using combined pulse oximetry and murmur screening in a cohort of 801,836 infants in Shanghai also showed high sensitivity and specificity for major and critical CHD [37].

Building fetal echocardiography screening protocols in low- and middle-income countries can be difficult [38]. Previously published work suggests that there may be a lack of knowledge about the need for referral for a fetal echocardiogram in Southeast Asia [38], as well as a lack of comprehensive multi-disciplinary teams and infrastructure to facilitate the transition from prenatal to postnatal care. As another barrier, prenatal detection may be much more costly than postnatal pulse oximetry screening, resulting in significant barriers in some countries. Other studies suggest cost benefits of prenatal detection.

Despite obstacles to prenatal CHD diagnosis in developing parts of the world, studies have demonstrated feasible implementation of fetal cardiac programs in low- and middle-income countries, with reasonable sensitivity and specificity [39]. Moreover, incorporation of such programs has led to better outcomes, including improved preoperative clinical status of neonates with CHD requiring surgery [40,41]. It stands to reason that ultrasound-based fetal cardiac screening may be a valuable strategy even in resource-limited settings.

Although fetal echocardiography in high-income countries often entails expensive ultrasound machines yielding detailed, high-resolution images, portable ultrasound technology is becoming an increasingly recognized approach to enable broader access to care. In obstetrics evaluations, handheld point-of-care ultrasound (POCUS) has been shown to be highly reliable when compared to conventional devices in the detection of fetal cardiac activity [42]. Future studies are needed to evaluate the utility of portable devices in more detailed fetal cardiac screening.

## 9. Access to Care and the Utilization of Novel Geospatial Mapping Techniques

Studies have shown variation in the sensitivity and specificity of fetal echocardiography based on the healthcare setting and provider [43]. During the pandemic, the use of innovative mapping techniques to identify outbreaks of COVID-19 were important in hotspot detection. The same types of tools can be used to study patterns of other more chronic diseases like cancer, which may also be environmental. The use of these techniques in determining rates of detection of CHD is relatively new but may facilitate efforts to ensure access to care. Clustering of disease has previously been described in pediatric illnesses [44] and as a potential tool for planning access to care [45]. Neighborhood influences on CHD have not been well-delineated, though the Baltimore-Washington Infant study did identify environmental exposures that may be associated with certain forms of CHD [46]. Therefore, specific regions of the world may both have an increased incidence of disease and the least access to advanced imaging technologies. In addition, maternal health conditions such as obesity and poorly controlled diabetes are linked to increased burden of CHD.

## 10. Emerging Technologic Solutions to Improve Fetal Echo Screening: Telehealth and Artificial Intelligence

**Figure 1:**
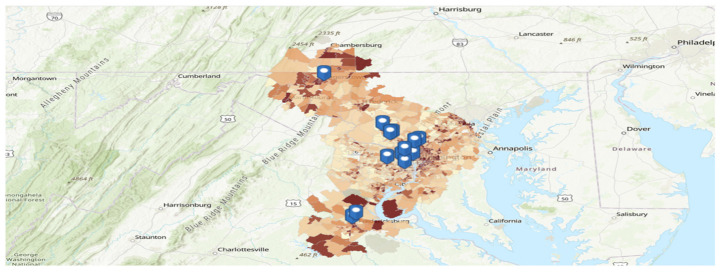
Quality improvement study plotting the location of outreach screening clinics (blue ovals) in relation to percentage of the census tract living below the poverty level. Darker shaded areas indicate the highest percentage of the population below the census tract. This identified that outreach is associated with increased density of obstetric subspecialists in more urban areas.

With increasing awareness of regions of missed prenatal detection, paralleling low-density obstetric regions or ‘deserts’, it may be possible to improve detection through telehealth outreach [47,48] to overcome limitations in current screening (see Figure 1). Standardized guidelines have made efforts to improve rates of prenatal diagnosis among all types of ultrasound testing, including anatomic ultrasound screening and formal fetal echocardiography performed by subspecialists [49]. Additionally, the feasibility of implementing telehealth with similar accuracy to in-person visits has been shown [48,50]. Telehealth outreach can be developed once important areas for outreach are identified.

While promise lies in technologies such as artificial intelligence (AI) [51], it will be critical to identify areas with decreased access where these technologies must be implemented, and where there may be barriers to the uptake of these technologies [52]. Artificial intelligence is already impacting cardiac diagnosis on a global level through AI-aided tools for auscultation [53], ultrasound interpretation [54], and ECG interpretation [55]. A multicenter study of 1413 children in Finland showed the potential for an AI-guided stethoscope to distinguish innocent murmurs from true disease [56]. Additional studies showed potential to classify CHD subtypes using electronic stethoscopes [57].

Existing barriers include learning time and the cost of new equipment. Low- and middle-income countries may be less likely to have ultrasound systems where these technologies can be used. Novel miniaturized handheld devices with AI capability, like those used in rheumatic heart disease scanning, may provide the solution [58]. Additionally, there may be an unknown component of infant death due to undiagnosed CHD (stillbirths or neonatal deaths where no autopsy is performed). Rapid advances in AI may improve prenatal detection in those who receive anatomical scans at centers that can afford the new technologies. However, it also may augment disparities if those technologies may not be available in developing countries for a number of years. Births by midwives in the United States are rising, reaching 12% in 2021 [59]. In many parts of the world, for example in Scandinavian countries, midwives are present in delivery in as many as 98.7% of deliveries [60]. A number of benefits may exist, and it is also important to understand the referral patterns for cardiac screening in these deliveries. Less than 2% of deliveries in the United States occur at home [61]. Around the world, more than 2 million women may deliver with no one present [62]. Globally, the prevalence of home birth is 28% [63], but some countries have very high rates of 50–78%, particularly in East Asia and Africa (Chad, Ethiopia, Niger, Yemen). The Netherlands has historically had a very high prevalence of home births, and since 2019 home births have remained at levels of 23–26% [64]. Home births and deliveries with no one present provide additional challenges to pulse oximetry screening for CHD.

## 11. The Dual Roles of Pulse Oximetry Screening and Fetal Echocardiography

Given variable availability of fetal cardiology expertise within and between regions, screening for critical and severe CHD is best done in an approach that partners prenatal and postnatal testing with a specific plan for each country that reflects the patient population, availability and skills of providers, and access to technology. Even in the most well-resourced areas, we are far from timely universal prenatal detection. The sensitivity of obstetrical cardiac ultrasound screening and fetal echocardiography is not anticipated to reach 99% globally for many years. Additionally, there may be false positives or misdiagnoses as well as lesions that cannot be detected, such as coarctation of the aorta or pulmonary vein anomalies. Together, using technologies in both the prenatal and postnatal period can be paired to achieve close to universal detection of CHD for infants in the perinatal period.

## Data Availability

No new data were created or analyzed in this study. Data sharing is not applicable to this article.
